# Sodic Soil Properties and Sunflower Growth as Affected by Byproducts of Flue Gas Desulfurization

**DOI:** 10.1371/journal.pone.0052437

**Published:** 2012-12-21

**Authors:** Jinman Wang, Zhongke Bai, Peiling Yang

**Affiliations:** 1 College of Land Science and Technology of China University of Geosciences, Handian District, Beijing, People’s Republic of China; 2 Key Laboratory of Land Consolidation and Land Rehabilitation Ministry of Land and Resources, Beijing, People’s Republic of China; 3 College of Hydraulic and Civil Engineering, China Agricultural University, Handian District, Beijing, People’s Republic of China; Kansas State University, United States of America

## Abstract

The main component of the byproducts of flue gas desulfurization (BFGD) is CaSO_4_, which can be used to improve sodic soils. The effects of BFGD on sodic soil properties and sunflower growth were studied in a pot experiment. The experiment consisted of eight treatments, at four BFGD rates (0, 7.5, 15 and 22.5 t ha^−1^) and two leaching levels (750 and 1200 m^3^ ha^−1^). The germination rate and yield of the sunflower increased, and the exchangeable sodium percentage (ESP), pH and total dissolved salts (TDS) in the soils decreased after the byproducts were applied. Excessive BFGD also affected sunflower germination and growth, and leaching improved reclamation efficiency. The physical and chemical properties of the reclaimed soils were best when the byproducts were applied at 7.5 t ha^−1^ and water was supplied at 1200 m^3^·ha^−1^. Under these conditions, the soil pH, ESP, and TDS decreased from 9.2, 63.5 and 0.65% to 7.8, 2.8 and 0.06%, and the germination rate and yield per sunflower reached 90% and 36.4 g, respectively. Salinity should be controlled by leaching when sodic soils are reclaimed with BFGD as sunflower growth is very sensitive to salinity during its seedling stage.

## Introduction

Desulfurization technologies reduce SO_2_ emissions in the flue gas of coal combustion, but they also produce large amounts of byproducts from flue gas desulfurization (BFGD) [1]. Substantial evidence exists that these BFGD can, with proper use, be valuable for soil reclamation, but improper use can be detrimental to the soil quality and to the environment when they enter the water and soil system via rainfall and surface runoff [2]–[4].

Typical sodic soils contain an excess of exchangeable sodium (ES) in the soil colloids, and the soluble carbonates are in the form of Na_2_CO_3_ and NaHCO_3_. The pH value, sodium adsorption ratio (SAR) and exchangeable sodium percentage (ESP) are greater than 8.5, 13 and 15 respectively, and the electrical conductivity of the saturated paste (EC_sat_) is less than 4.0 dS m^−1^. The key to reclamation is to remove the ES and replace it with more favorable calcium ions in the topsoil [5]. Historically, sodic soils have been reclaimed with gypsum (CaSO_4_·2H_2_O), but gypsum has become unpopular because of its high price. The main character and components of BFGD are similar to those of gypsum, and they also contain sufficient amounts of the minerals that are necessary to crops, such as Ca, S and so on. At present, most BFGD are discarded, primarily into landfills, using up land resources and with increasing disposal costs [6]. Interest has been growing in using BFGD to reclaim sodic soils. Recent research has shown that BFGD can appreciably decrease soil pH and ESP, with no environmental impacts from trace metals, and significantly increase germination rates and crop outputs [2], [4], [7]–[10]. However, research on reclaiming sodic soils with BFGD is still in an elementary stage, and in China such reclamation is used only on a small scale (e.g., in Kangping county of Liaoning Province, Tumochuan county and Wulateqian county of Inner Mongolia, and on the Hetao plain of Ningxia Province).

Some studies have indicated that different crops have different degrees of sensitivity to salinity at different growth stages [11]–[12]. The tolerance of salinity was relatively high for the crops that were sensitive to salinity in the seedling stage, but relatively low for the crops that were not sensitive to salinity [11]. When BFGD are used in the reclamation of sodic soils, their high content of soluble salts may adversely affect plant growth, as they reduce the osmotic potential of plant roots, even though they are a good source of CaSO_4_ [12].

Thus, it is necessary to determine a suitable application rate for BFGD that allows crops to tolerate not only sodicity, but also salinity. The objective of this research was to analyze the responses of sodic soil properties and sunflower growth to BFGD and to select an application rate and mode of application that would simultaneously control sodicity and salinity.

## Materials and Methods

### Physical and Chemical Properties of Soils and BFGD

The soil tested was sampled from the Changsheng Experimental Station of Baoyannur League Institute of Water Resources in the northwest of China (N 40°20′, E 108°31′). The soil had typical characteristics of sodic soil, that is, a high pH and exchangeable sodium percentage, and low hydraulic conductivity. The soil texture is clay, and its physical and chemical properties are listed in Table 1. The soil was air-dried, crushed and passed through a 2-mm sieve before the pot experiments. The BFGD tested was from Huaneng Power International, Inc. The particle size of the BFGD is between 20 µm and 80 µm. The CaSO_4_ content in BFGD was 89.8%, the amount of moisture in BFGD was almost 10.1% (Table 2), and the concentrations of pollution elements in the BFGD were far below the tolerance limits regulated by the Control Standards for Pollutants in Fly Ash for Agricultural Use (GB8173-87) and the Control Standards for Pollutants in Sludge for Agricultural Use (GB4284-84) [13]–[14].

**Table 1:** Physical and chemical properties of soil.

Property	Parameter	Value	Property	Parameter	Value
Particle size distribution	2.0–0.02 mm (%)	23	Chemical	ES(cmol kg^−1^)	3.85
	0.02–0.002 mm (%)	35		CEC(cmol kg^−1^)	8.98
	<0.002 mm (%)	42		ESP(%)	42.85
Physical	Bulk density(g cm^−3^)	1.48		EC(dS m^−1^)	2.15
	HC (cm min^−1^)	2.51×10^−5^		pH	9.15
Soluble cations	Na^+^(cmol L^−1^)	0.81	Soluble anions	HCO_3_^−^(cmol L^−1^)	0.16
	K^+^(cmol L^−1^)	0.01		CO_3_^2−^(cmol L^−1^)	0.04
	Ca^2+^(cmol L^−1^)	0.05		SO_4_^2−^(cmol L^−1^)	0.36
	Mg^2+^(cmol L^−1^)	0.03		Cl^−^(cmol L^−1^)	0.34

Note: Value of the mean of three replicates; HC is hydraulic conductivity.

**Table 2:** Component and properties of the BFGD.

pH	Density (g·cm^−3^)	Free water (%)	CaSO_4_·2H_2_O (%)		CaSO_4_·1/2H_2_O (%)		CaCO_3_ (%)
5.90	1.02	10.10	89.8		0.20		5.55
**Cd (mg·kg^−1)^**	**Cr (mg·kg^−1^)**	**As (mg·kg^−1^)**	**Se (mg·kg^−1^)**	**Ni (mg·kg^−1^)**	**Cu (mg·kg^−1^)**	**Hg (mg·kg^−1^)**	**Pb (mg·kg^−1^)**
0.01	83.4	5.04	4.24	21.35	83.26	0.32	99.38
5^a^	250^a^	75^a^	15^a^	200^a^	250^a^	5.0^b^	250^a^

Note: ^a^Control Standards for Pollutants in Fly Ash for Agricultural Use (GB8173–87);

b Control Standards for Pollutants in Sludge for Agricultural Use (GB4284–84).

### BFGD Application Rate and Experimental Design

The application rate of the BFGD was determined using a modified method based on gypsum application rates [15]–[16]. It was calculated according to the relative content of calcium in BFGD and gypsum as follows.
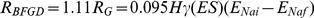
(1)where 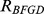 is BFGD application rate in t ha^−1^; 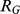 is the gypsum application rate in t ha^−1^; 1.11 is the modified coefficient, which was determined by comparing the content of CaSO_4_ in gypsum and BFGD; H is the soil depth to be reclaimed in cm, 20 cm was used in the research; 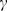 is the soil bulk density in g cm^−3^, which is 1.48 g cm^−3^ according to Table 1; 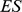 is the exchangeable sodium in cmol kg^−1^, which is 3.85 cmol kg^−1^ according to Table 1; 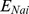 is the initial exchangeable sodium fraction, which is 1; and 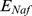
is the desired final exchangeable sodium fraction, 0.1 was used in the research. The BFGD application rate was 9.75 t ha^−1^, based on the initial properties of the sodic soil tested.

The pot experiments were carried out in the experimental field of China Agricultural University (N 39°48′, E116°28′) using sunflower G101. The pots were 23 cm in height, 19 cm in bottom diameter and 25 cm in top diameter, and were filled with 12.31 kg of dried soil, 3 g urea and a measured amount of BFGD. The soil bulk density in the pots was 1.48 g cm^−3^, and the BFGD was mixed with the soil of 0–10 cm depth prior to packing. The irrigation cycle is 12 days, and aluminum containers were placed in the bottom of each pot to collect leachate. On June 8, 2004, 20 sunflower seeds were sown in each pot and the plants grew under natural conditions. The sunflowers were harvested on September 21, after 106 days of growth. The leachate were collected four time, the dates were May 21, June 5, July 4 and July 10 respectively; Three soils were sampled with an auger at soil depths of 0 to 10 cm and 10 to 20 cm on June 30, August and September 9 respectively.

The experiment consisted of eight treatments (Table 3), at four BFGD rates (0, 7.5, 15 and 22.5 t ha^−1^; or 0, 28.5, 57.0 and 83.6 g per pot) and two leaching levels (750 and 1200 m^3^ ha^−1^; or 3 l per pot and 4.5 l per pot). There were eight replicates for each treatment.

**Table 3:** Experimental design.

Experimental treatments	T1	T2	T3	T4	T5	T6	T7	T8
BFGD rate (t ha^−1^)	0	0	7.5	7.5	15	15	22.5	22.5
Leaching water (m^3^ ha^−1^)	750	1,200	750	1,200	750	1,200	750	1,200

### Analytical Methods and Statistical Analyses

The samples were air-dried after the removal of the plant roots and passed through a 1-mm sieve. The EC, pH, soluble anions, and soluble cations were measured using 1∶5 water extracts. The soluble cations were measured using an atomic absorption spectrophotometer; soluble anions were determined by anion chromatography; exchangeable cations were determined in 1 M ammonium acetate (pH = 7) extract, and cation exchange capacity was determined by the removal of ammonium ions by distillation following this extraction and washing with 96% alcohol [17]. Na and K were determined by flame emission spectroscopy in the extract, and Ca and Mg were determined by atomic absorption spectrophotometer. Soil pH was determined with the glass electrode method. The salt content was measured using a 1 cm conductivity cell, dip-type probe. Saturated hydraulic conductivities (HC) were determined by using cutting ring and calculated by using Darcy’s law. Particle size distribution was determined with the hydrometer method.

Measurements of physical and chemical items were duplicated, and there were three replicates for the chemical analysis. Standard errors of the means of the three samples from each treatment were calculated. The variation among the treatments was analyzed with STATISTICA software (StatSoft, Inc; USA).

## Results

### The Effects of BFGD and Leaching on Sunflower Germination

**Figure 1:**
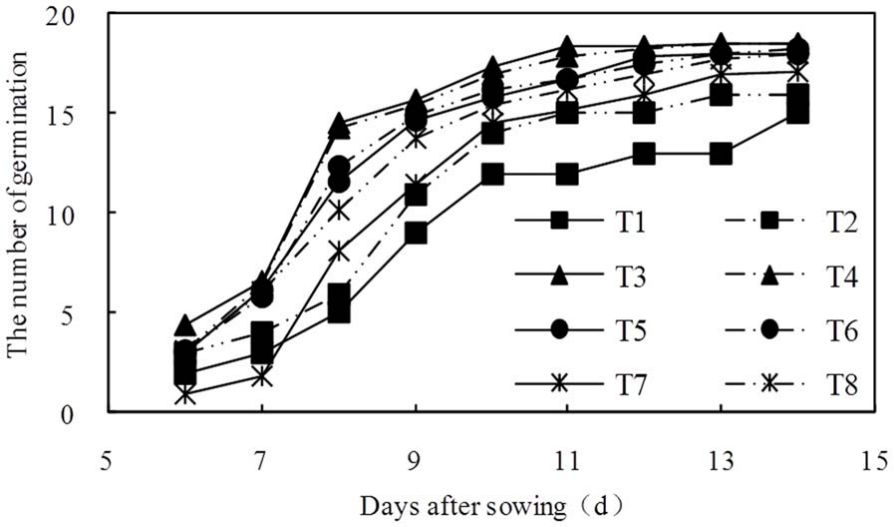
Variation of sunflower germination with days after sowing.

**Figure 2:**
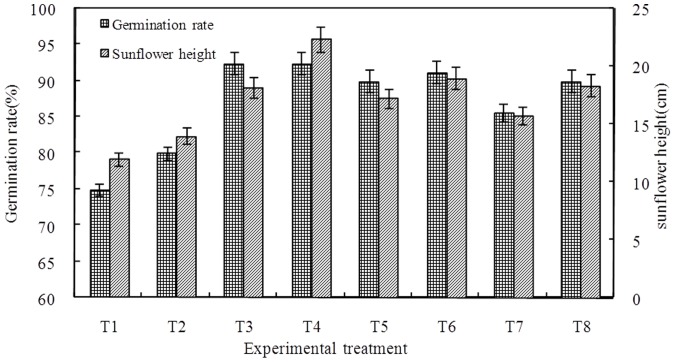
Germination rate and sunflower height in the seedling stage.

All of the sunflowers germinated between the 6th and 14th day after sowing (Fig. 1). The germination of the sunflower plants occurred in two stages. The quantity of germination was greater in the first stage, and these time slots were 6–10 d, 6–8 d, 6–9 d and 6–9 d with 0 t·ha^−1^, 7.5 t ha^−1^, 1 5 t ha^−1^ and 22.5 t ha^−1^ BFGD rates, respectively. The quantity of germination was relatively less during the second stage, and these time slots were 10–14 d, 8–14 d, 9–14 d and 9–14 d, respectively. The different BFGD rates and leaching levels had different effects on the germination rate (*P*<0.05; Fig. 2). The germination rate with low BFGD rates was higher than that of plants with high BFGD rates, and the germination rate with high leaching levels was higher than that of plants with low leaching levels. It can be concluded that BFGD both increased and accelerated the germination, but an excessive BFGD rate also suppressed the germination and delayed the time of the germination.

**Figure 3:**
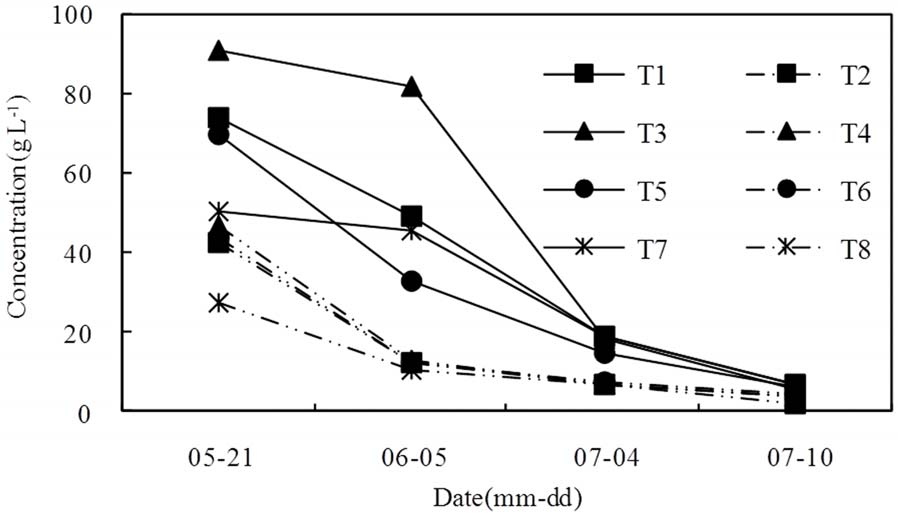
Variation of leachate concentration.

**Figure 4:**
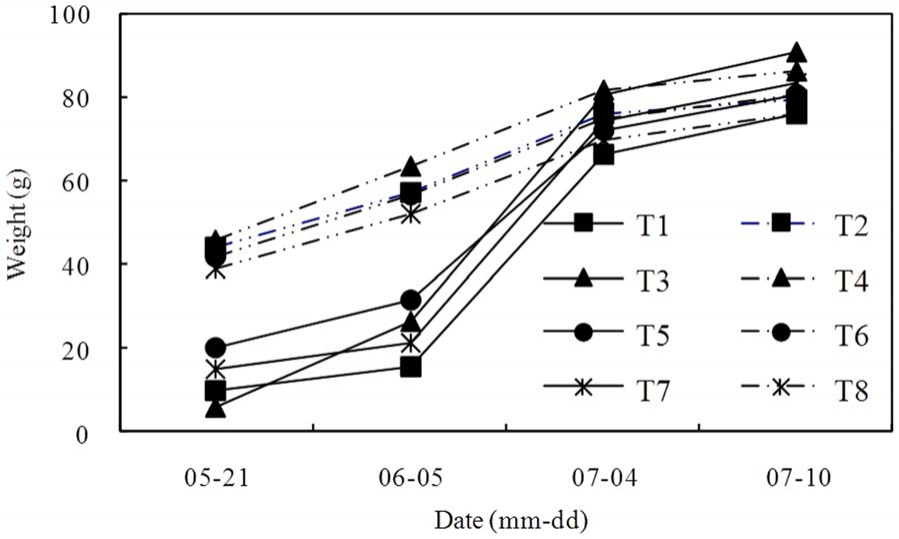
Buildup of salt content of leachate.

The variation in leachate concentration and the buildup of the salt content of the leachate are shown in Figs. 3 and 4, respectively. The leachate concentration of the high leaching level was lower than that of low leaching level, and the salt content was also different because of variation in the volume of leachate among different treatments. The salt content of the high leaching level gradually decreased, whereas the salt content of the low leaching water decreased after an initial increase. The accumulated salt content of the leachate was basically the same in all treatments. Although the total salt content of the leachate was basically equal, the effects were not equal as the time that the salt was held in the soil varied and was higher in some treatments during the seedling stage of the sunflowers. Therefore, the growth of the sunflowers varied. The sunflower heights with different treatments in the seedling stage are shown in Fig. 2. The height of plants treated with high leaching water was higher than plants treated with low leaching water, and the height of sunflower plants with a low BFGD rate was higher than that of plants with a high BFGD rate.

### The Effects of BFGD and Leaching on Sodic Soil Properties

**Figure 5:**
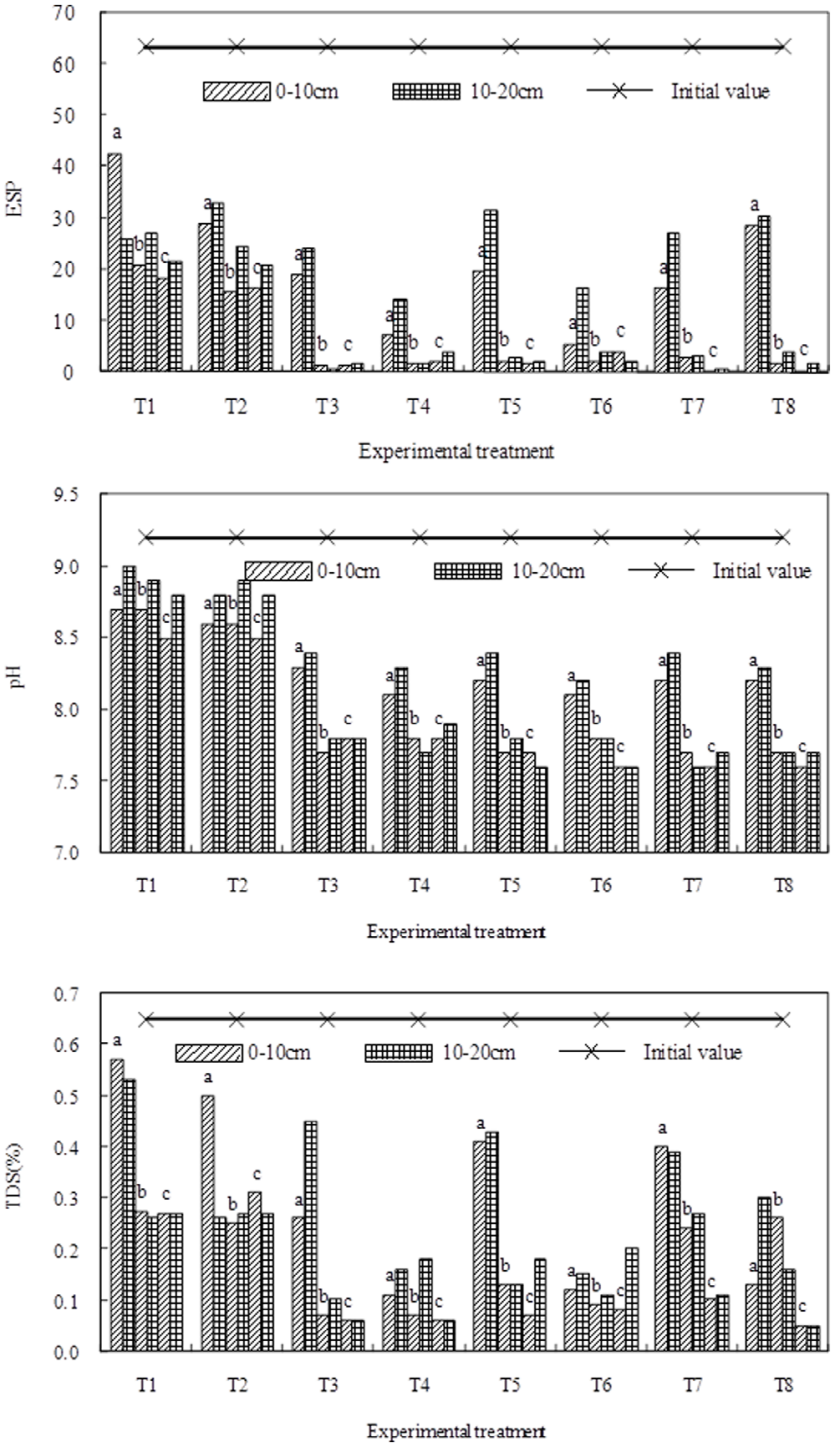
The ESP, pH and TDS of the soil as affected by the BFGD and leaching (a) June 30; b) August 13; c) September 9).

The ESP, pH and TDS for different treatments were compared with each other on June 30, August 13, and September 9 and the results are depicted in Fig. 5. The ESP, pH and TDS significantly decreased at soil depths of 0 to 10 cm and 10 to 20 cm after applying BFGD and leaching. At the 0–10 cm soil depth, the pH, ESP, and TDS of soils treated with a low BFGD rate and a high leaching level were lower than in the 10–20 cm depth of soils treated with a high BFGD rate and a low leaching level. The physical and chemical properties of the reclaimed soil were best when the by-products were applied at 7.5 t·ha^−1^ and the water was supplied at 1200 m^3^ ha^−1^; under these conditions the soil pH, ESP, and TDS decreased from 9.2, 63.5 and 0.65% to 7.8, 2.8 and 0.06% respectively.

### The Effects of BFGD and Leaching on Sunflower Growth

**Figure 6:**
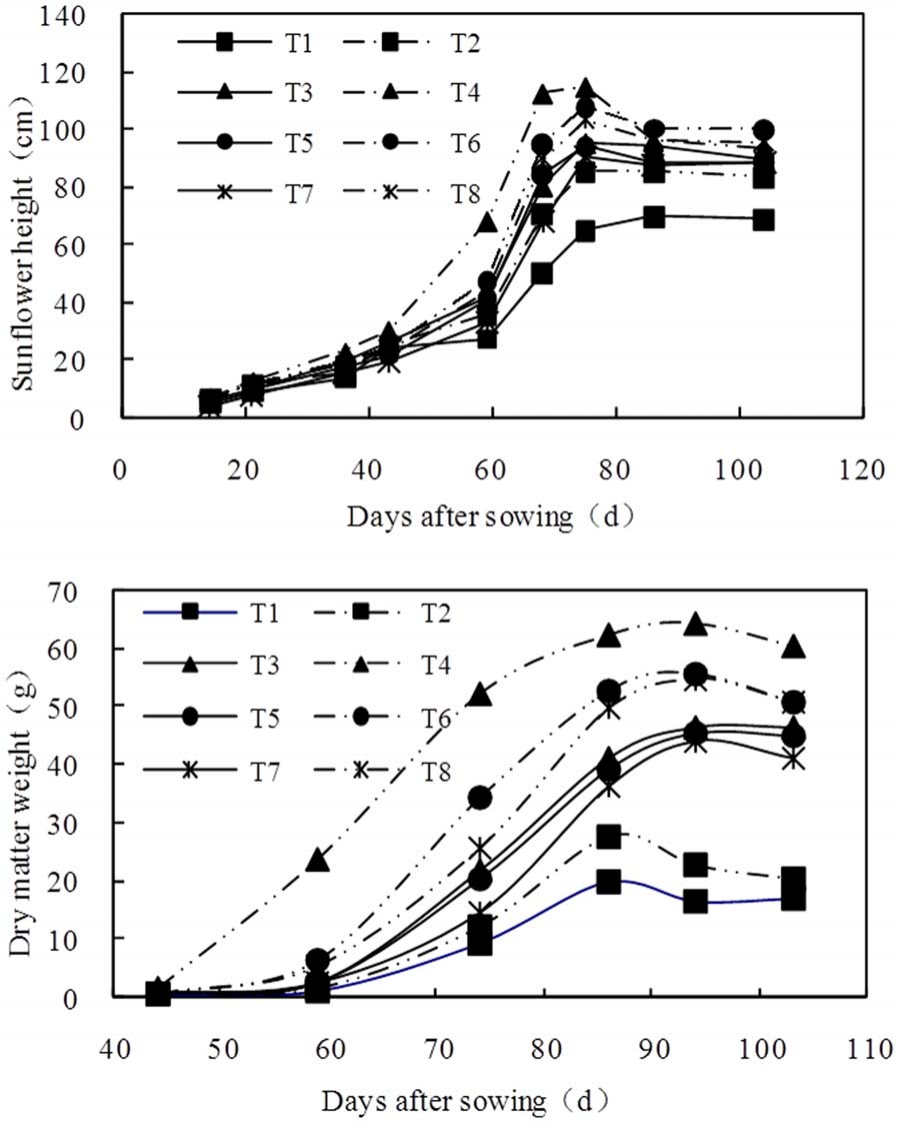
Variation of sunflower height and dry matter weigh with days after sowing.

The variations in sunflower height and dry matter weight are shown in Fig. 6. The sunflower height and dry matter weight were markedly increased by the application of the byproducts and leaching. Height and dry matter weight were greatest when the byproducts were applied at 7.5 t ha^−1^ and water was supplied at 1200 m^3^ ha^−1^. So, BFGD application made good initial sunflower stands possible and enhanced sunflower growth, presumably increasing leaching through root channels and the exchange of sodium to a deeper soil depth.

### The Response of Sunflower Yield to BFGD and Leaching

**Figure 7:**
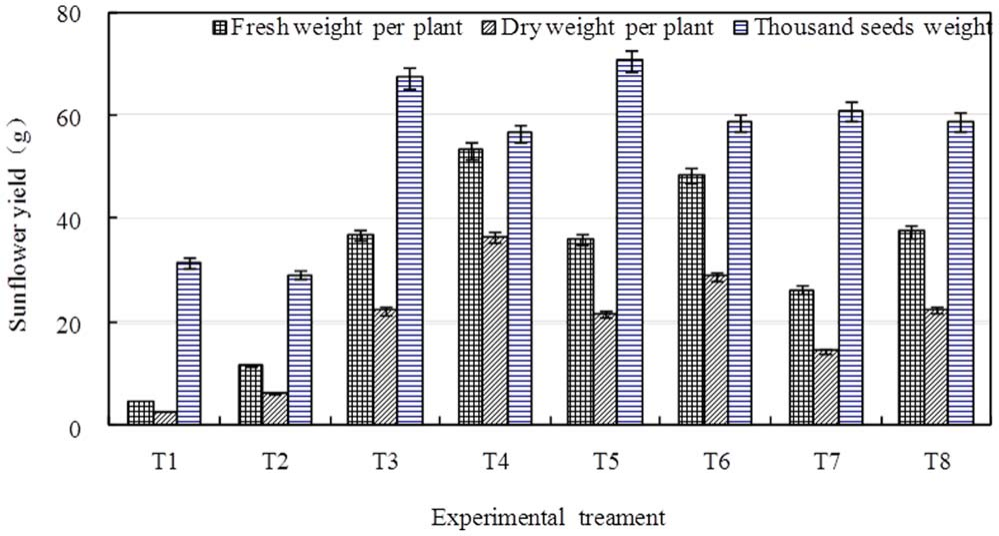
Variation of sunflower yield.

Fig. 7 compares the effects of BFGD and leaching on the yield per sunflower and on the one thousand seeds weight. The output of the sunflower had no positive correlation with BFGD rate. BFGD could increase the output of the sunflowers when they were applied in the right amount, but the output of the sunflowers decreased if the applied amount of BFGD increased beyond the sunflowers peak value output. The output of sunflowers under the high leaching condition was higher than under the low leaching condition. The sunflower output was best when the byproducts were applied at 7.5 t ha^−1^ and water was supplied at 1200 m^3^ ha^−1^; under these conditions the output of one sunflower could reach 36.4 g. The variation in the one thousand seeds weight was not the same as the variation in the output of one sunflower; the one thousand seeds weight at the high leaching level was lower than that at the low leaching level.

The effects of BFGD and leaching on the yield per sunflower were analyzed with progressive regression analysis using STATISTICA software. The regression equation was as follows.
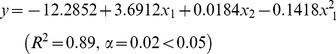
(2)where 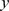 is the yield per sunflower in g; 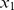 is the application rate of BFGD in t ha^−1^; and 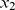 is the amount of leaching water in m^3^ ha^−1^.

## Discussion

### Mechanism of the Effect of BFGD on Sodic Soil and Sunflower Growth

The dispersion of clay in sodic soils can be reduced three-fold when soil pH decreases from 9 to 7, and soil pH is an important factor in managing dispersive soils [18]. The by-products clearly reduced the ESP, pH, TDS and clay dispersion and thus improved the physical properties of the soil. Through the intervention of calcium, BFGD can reduce the electric potential produced by the mutual exclusion of negative charges in the soil colloids surface, promote the formation of a soil aggregate structure, raise soil water holding capacity, lower soil bulk density, increase soil porosity, improve the growth environment of root systems, increase the germination rate of sunflowers, and promote the growth of sunflowers [5], [19]. So, BFGD application in poorly structured soils helped crop establishment and increased crop growth [20]–[21].

### Soil Salinity Control in the Process of Reclaiming Sodic Soil with BFGD

With the application of BFGD the salt content of the soil will increase. As crops are very sensitive to salinity, especially in the seedling stage, the salt in the soil must be promptly removed [11]–[12]. In this study, although the total salt content of the leachate was basically equal in all treatments, the residence time of salinity in the soil varied, which led to different effects. The improvement effects had no positive correlation with BFGD rate. The effect of a high leaching level was better than that of a low leaching level. Initially, the increase of BFGD application rate resulted in soil improvement, but beyond a certain application rate threshold, the improvement effects began to decrease. Thus, when reclaiming sodic soil with BFGD, the soil salinity needs to be reasonably controlled. Under field conditions the salts should be immediately removed by constructing good drainage conditions. On the other hand, microbial activities are very important to the reclamation of sodic soils and can accelerate the improvement of soil structure [22]–[23]. So, the regulation of microbial activity is also our future research focus.

### The Best BFGD Application Rate

The relationship between the application rate of BFGD and sunflower yield per plant fits a parabola shape. The relationship is:
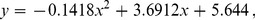
(3)where 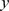 is the yield per sunflower in g and 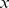 is the application rate of BFGD in t ha^−1^.

The above equation was differentiated and the following formula was obtained.
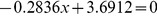
(4)


It was calculated that the yield per sunflower was the highest when the application rate of BFGD was 13 t·ha^−1^. This application rate is 1.34 times the amount of the theoretical calculations. The best application rate was obtained in the pot experiment conditions, so the higher application rates might be used in field conditions due to the limitations of various factors [2], [8].

### Conclusions

The following conclusions can be drawn from our findings.

1. Application of the by-products from flue gas desulfurization is an efficient method to reclaim sodic soil. The germination rate and output of sunflower plants significantly increased, and the exchangeable sodium percentage (ESP), the pH, and the total dissolved salts (TDS) decreased. However, excessive amounts of BFGD also negatively affected germination and growth of sunflowers.
2. Leaching improves the efficiency of the reclamation. Salinity should be controlled by leaching when sodic soils are reclaimed with BFGD, as the sunflower growth is very sensitive to salinity in the seedling stage.
3. The physical and chemical properties of the soil with a value of pH = 9.2 and ESP = 63.5 were best when the by-product was applied at 7.5 t ha^−1^ and water was supplied at 1200 m^3^ ha^−1^. Under these conditions the germination rate and yield per sunflower could reach 90% and 36.4 g, respectively. Therefore, a reasonable application rate should be selected when BFGD are used to reclaim sodic soils.
